# Sequence diversity on four ORFs of citrus tristeza virus correlates with pathogenicity

**DOI:** 10.1186/1743-422X-6-116

**Published:** 2009-07-30

**Authors:** Lisset Herrera-Isidrón, Juan Carlos Ochoa-Sánchez, Rafael Rivera-Bustamante, Juan Pablo Martínez-Soriano

**Affiliations:** 1 Centro de Investigación y de Estudios Avanzados del Instituto Politécnico Nacional, Campus Guanajuato, Km. 9.6 Libramiento Norte, Carretera Irapuato-León, 36821 Irapuato, Guanajuato, Mexico

## Abstract

The molecular characterization of isolates of citrus tristeza virus (CTV) from eight locations in Mexico was undertaken by analyzing five regions located at the opposite ends of the virus genome. Two regions have been previously used to study CTV variability (coat protein and p23), while the other three correspond to other genomic segments (p349-B, p349-C and p13). Our comparative nucleotide analyses included CTV sequences from different geographical origins already deposited in the GenBank databases. The largest nucleotide differences were located in two fragments located at the 5' end of the genome (p349-B and p349-C). Phylogenetic analyses on those five regions showed that the degree of nucleotide divergence among strains tended to correlate with their pathogenicity. Two main groups were defined: mild, with almost no noticeable effects on the indicator plants and severe, with drastic symptoms. Mild isolates clustered together in every analyzed ORF sharing a genetic distance below 0.022, in contrast with the severe isolates, which showed a more disperse distribution and a genetic distance of 0.276. Analyses of the p349-B and p349-C regions evidenced two lineages within the severe group: severe common subgroup (most of severe isolates) and severe divergent subgroup (T36-like isolates). This study represents the first attempt to analyze the genetic variability of CTV in Mexico by constructing phylogenetic trees based on new genomic regions that use group-specific nucleotide and amino acid sequences. These results may be useful to implement specific assays for strain discrimination. Moreover, it would be an excellent reference for the CTV situation in México to face the recent arrival of brown citrus aphid.

## Background

Citrus tristeza virus (CTV) is a destructive pathogen that causes the most important disease of citrus. CTV is phloem-limited and naturally transmitted by several species of aphids in a semipersistent mode and by the use of infected propagative budwood [1].

CTV infects almost all citrus species, including hybrids and relatives, causing different range of symptoms depending on the host and virus strain. The most common symptoms are: quick decline (QD) or death of most citrus species propagated on the sour orange (*Citrus aurantium *L.) rootstock; stem pitting (SP) of the scion that reduces the yield and fruit quality of some citrus varieties regardless of the rootstock; and seedling yellows which is seen on sour orange, lemon (*Citrus lemon *(L.) Burn. f.) and grapefruit (*Citrus paradisi *Macf.). Strains that not elicit symptoms on commercial citrus hosts have been named as mild isolates [2].

Citriculture represents a very important activity in Mexico where 517,000 has. are grown in several agroecological regions yielding 7.1 million tons of fruit [3]. CTV was first detected in the Mexican State of Tamaulipas in 1983. Extensive survey in the major citrus growing regions indicated (up to 2006) that CTV incidence was low (< 1%) in other 17 States of Mexico. Because of the random pattern of the infected trees, CTV infection was probably propagated in Mexico by the use of infected budwood, rather than by aphid vectors [4]. In general, the trees remain asymptomatic, without declining suggesting a possible prevalence of mild strains. However, Mexican citrus industry is now facing a real and immediate threat due to: i) the recent introduction to Mexico of the most efficient vector of CTV, commonly called brown citrus aphid (BCA), *Toxoptera citricida *and ii) the predominance of sour orange as rootstock (the most susceptible to CTV) in almost the totality of the citrus groves.

In early 2000, *T. citricida *was first found in Quintana Roo, Mexico apparently spreading from Belize. Recently it reached the citrus regions of the Mexican states of Campeche, Yucatán, Veracruz and Tabasco. Several favorable conditions exist in Mexico for the spreading of BCA. This could facilitate the displacement of migrant winged insects to new locations.

Despite the importance of CTV, there is a lack of knowledge about the genetic diversity of CTV in Mexico. Therefore, the objective of this study was to determine the molecular variability of CTV isolates collected in eight of the major citrus growing areas in Mexico, before the spreading of the BCA.

## Results

### Biological characterization of the CTV isolates

Symptoms of CTV isolates varied in their severity and ranged from a severe SP observed in the isolates Mx-Tam and Mx-BC (Mexican lime on *C. macrophylla*) to those as Mx-Yuc and Mx-QR, which showed a mild vein clearing in Mexican lime. Incipient SP and vein clearing were observed for Mx-Mich, Mx-NL, Mx-Col, Mx-Ver and Mx-QR. When sour orange was used as rootstock, a gradual declining occurred for the Mx-Tam isolate associated to pinholing or honeycombing below the bud union.

### Phylogeny and genetic variability in CTV isolates

The nucleotide sequences of five regions of the CTV genome were determined. They are situated at the opposite ends covering around the 20% of the CTV genome. These DNA fragments include two fragments of the p349 ORF located at the 5'end of the genome: p349-B (nucleotides 1460 to 2350) and p349-C (nucleotides 6870 to 7900) and the ORFs CP, p23 and p13 (located at the 3' end).

Alignments of these genomic regions indicated that variations at nucleotide level were simple nucleotide changes. Overall similarities for the five regions among CTV Mexican isolates ranged from 71.2 to 100% at the nucleotide level; however, the sequences of these strains clustered into two groups that were highly homologous (92.1–100% identical nucleotides and 92.9–100% identical amino acid) such as Mx-Mich, Mx-Ver, Mx-QR, Mx-NL, Mx-Yuc and Mx-Col and less homologous sequences (71.2–96.7% identical nucleotides and 71.6–96.4% identical amino acid) such as Mx-Tam and Mx-BC (data not shown).

The neighbour-joined method of Clustal × program was used to generate phylogenetic trees for each ORF including the following full length CTV sequences: T36 [5] and T30 from Florida [6], VT from Israel [7], T385 from Spain [8], SY568 from California [9] and NUagA from Japan [10]. In order to compare our five amplified regions as individual ORFs, sequences from regions p349-B and p349-C were combined and named as p349-B/C.

**Figure 1:**
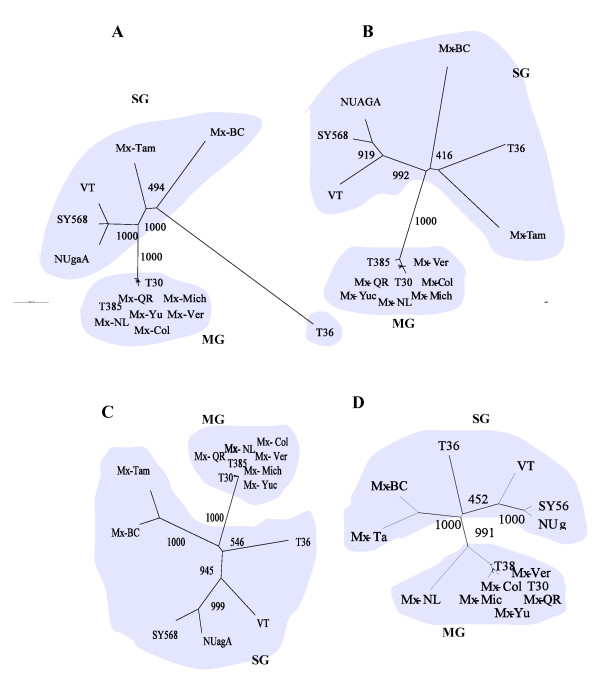
**Unrooted phylogenetic trees of the genomic regions p349-B/C (A), CP (B), p13 (C) and p23 (D) for 14 CTV isolates**. The virus isolates that fell in separated groups are shaded and labeled with the group name (MG, mild group; SG, severe group). Trees were constructed by neighbor-joining method using nucleotide sequences aligned with Clustal × program and 1,000 bootstrap replications. Only the values over 400 are shown. The scale bar represents the number of nucleotide replacements per site: 0.01.

The phylogenetic trees were topologically similar for all the ORFs. A very consistent and defined cluster was observed with a bootstrapping over 990, which included the highly homologous Mexican isolates (Mex-QR, Mex-Mich, Mex-NL, Mex-Col, Mex-Ver and Mex-Yuc) as well as isolates T30 and T385 (figure 1). Despite the distant geographical origin of the isolates, they all induced mild symptoms on indicator plants and only mild stem pitting on the Mexican lime.

On the other hand, CTV Isolates Mex-Tam, Mex-BC, NUagA, VT, SY568 and T36, which induced severe symptoms, showed a more disperse phylogenetic distribution (figure 1). The comparative analysis revealed that both severe Mexican isolates Mx-Tam and Mx-BC, tended to be clustered away from the group formed by NUagA, VT and SY568, although not enough to be considered a separate cluster. Similar clustering was obtained using the predicted amino acid sequences (data not shown).

The calculated sequence diversity for the mild cluster was almost the same for the five analyzed regions ranging from 0.002 to 0.022. On the contrary, the severe cluster showed an increased diversity especially toward the 5'end of the genome (CP < p23 < p13 < p349-B < p349-C) (table 1).

**Table 1:** Average number of nucleotide distance between CTV isolate groups in different genomic regions.

CTV region	CTV group	D^a^
P349-B	Mild	0.008 ± 0.002
	Severe	0.233 ± 0.014
	All	0.143 ± 0.009
		
P349-C	Mild	0.008 ± 0.002
	Severe	0.278 ± 0.013
	All	0.170 ± 0.012
		
CP	Mild	0.003 ± 0.001
	Severe	0.066 ± 0.007
	All	0.054 ± 0.006
		
P13	Mild	0.002 ± 0.001
	Severe	0.094 ± 0.011
	All	0.068 ± 0.008
		
P23	Mild	0.022 ± 0.003
	Severe	0.086 ± 0.008
	All	0.078 ± 0.007

^a^D, nucleotide diversity (average number of nucleotide substitution per site between pair of sequence).

Interestingly, two observations could be emphasized for the p349 gene. First, the genomic regions p349-B and p349-C showed the greatest diversity (0.143 and 0.169, respectively), twice as much as the one for CP, p13 and p23. Second, the T36 isolate remained separated in the phylogenetic tree for p349-B/C region. When the genetic distances were calculated for the severe group excluding the T36 isolate, it showed little variation for CP, p13 and p23, but it diminished two-fold p349-B (0.233 to 0.143) and for p349-C (0.276 to 0.120) (data not shown). The marked increase in the genetic diversity given by the T36 isolate suggests the presence of two lineages within the severe group.

To further analyze this possibility, a 280-nucleotide fragment from p349-B region (nucleotides 3333 to 4000) was compared for 44 CTV isolates which nucleotide sequences and biological behaviors had been previously reported [11]. Topology of the phylogenetic trees constructed from sequences alignments (for nucleotide as well as for amino acid residues) was similar to those observed for the p349-B/C. In this case, the T36 isolate considerably diverged forming an additional cluster with other two severe CTV isolates (T734 and T346) (data not shown). Another phylogenetic tree confirmed the divergence of the selected CTV isolates into three groups according to the severity level: one contained the 30 isolates (mild group), a second contained the previously reported five severe isolates and other ten more (severe common group), and a third one contained severe isolates which were located in the same branch along with T36 isolate (severe divergent group). Within- and between-groups nucleotide diversity analyses indicated that all mild isolates had a very low average intra-group genetic diversity (0.013) while the severe groups showed values up to seven times higher than the mild isolates. The inter-group distances were always higher than within groups. The highest variability (0.633 and 0.584) was observed among the severe divergent group and the other two groups (mild and severe common) respectively, thus confirming that the severe divergent group represents a different CTV genotype (table 2).

**Table 2:** Within- and between-groups nucleotide diversities for the region spanning the last 400 nucleotides of p349-B region

	Mild	Severe common	Severe divergent
Mild	0.013 ± 0.003	0.136 ± 0.0014	0.633 ± 0.066
Severe common		0.099 ± 0.011	0.584 ± 0.06
Severe divergent			0.118 ± 0.016

Additionally, clones obtained from five Mexican isolates belonged to more than one group: mild and severe common groups. This was the case of severe isolates Mx-BC for p349-B and Mx-Tam for p349-C, and mild isolates Mx-Col, Mx-Mich and Mx-NL, illustrating the presence of different sequence variants in their gRNA populations. However, several rounds of amplification, cloning and sequencing were repeated for these isolates and only the consensus nucleotide sequences of the major variants were considered in the analyses.

Similarly, the CP and p23 sequences from 24 and 28 CTV isolates from the GenBank databases were compared, respectively. As a result, the spatial distributions in the phylogenetic trees for CP and p23 were very similar to the observed with only 14 isolates shown in the figure 1. The mild isolates were grouped in a single branch with a high intra-group homology greater than 98% while for the severe isolates this rank was smaller, between 80–85% (data not shown).

### Nucleotide and amino acid substitutions of the CTV variants

A further visual examination of the sequences alignments for all the isolates revealed group-specific features that can be used to classify isolates. Group-specific nucleotides could be identified for each ORF and many of them were located on regions p349-C (37.3%), p349-B (32.0%), followed by p23 (20.0%), CP (13.3%) and p13 (10.7%) (table 3). The majority of the sequence polymorphisms corresponded to single changes, and only three putative stretches of nucleotides were observed for the regions p349-C (positions 310–311 and 355–357) and p23 (positions 86–87).

**Table 3:** Nucleotide variation observed in CTV isolates^a^

	P349-B nuc. at position:															
	
	25	56	81	87	117	177	186	222	258	392	440	499	518	638	642	695
M	T	G	G	G	G	A	G	G	A	T	T	G	A	C	G	A
FSC	G	C	T	T	A	G	T	A	G	C	G	A	C	T	A	T
T36				C	T											C

																

	P349-B nuc. at position:								p349-C nuc. at position:							
		
	700	725	733	746	759	774	834	870		31	121	149	151	156	169	252

M	G	G	A	G	C	C	G	A		C	T	A	A	C	A	G
SC	A	A	G	A	T	T	A	G		T	G	S	B	W	K	A
T36	T	T										G	T	T	G	

																

	P349-C nuc. at position:															

	310	311	313	355	356	357	425	432	519	557	558	615	666	678	699	747

M	G	C	T	C	C	T	T	A	T	T	A	A	A	A	T	G
SC	T	T	M	R	R	V	S	S	R	S	G	Y	G	G	C	A
T36			G	G	A	C	C	C	A	G		C				

																

	p349-C nuc. at position:					CP nuc. at position:										
		
	797	809	854	962	1025	255		336	360		371	396		435	522	606

M	G	A	C	T	A	C		A	C		A	A		T	T	A
SC	A	G	K	C	G	T		G	W		T	T		C	C	G
T36			T						T							

																

	P13 nuc. at position:											P23 nuc. at position:				
			
	33	45	48	114	135	190	192	222	241	301		70	80	86	87	117

M	A	A	G	A	T	T	G	C	T	C		A	A	A	A	T
SC	G	G	A	G	A	C	A	T	G	A		G	C	G	T	R
T36																G

																

	P23 nuc. at position:															
	
		138	145		150	228		235	237		300	375		382	459	

M		T	C		G	C		C	C		G	A		T	A	
SC		C	T		A	T		T	G		A	T		A	G	
T36																

^a ^Nucleotide (nuc.) differences in five CTV genomic region among mild isolates (M), severe common isolates (SC) and T36 isolate. Nucleotide positions which resulted in amino acid residue changes are underlined. Only differences between T36 and SC are indicated for T36. S: C or G; M: A or C; B: C or G or T; W: A or T; K: G or T; R: A or G; V: A or C or G; Y: C or T.

Sequence alignments revealed that 85% of differential nucleotides could be classified in severe isolates (including T36 isolate) and the mild isolates. On the other hand, the 25% of the remainder polymorphisms distinguished the isolate T36 from the others, whether severe common or mild isolates. Most of these T36 typical changes were observed throughout the p349-B and p349-C genomic regions and only two of these types of changes were located at position 360 in the CP gene and at position 117 in the p23 gene (table 3). Finally, there were three nucleotide insertions at positions 153, 369 and 792 in the sequence of T36 for p349-C region (data not shown). Many of these nucleotide substitutions yielding amino acid changes were conserved within each group. From the 34 group-specific amino acid substitutions that were identified, 14 were located in the p349-B (table 4).

**Table 4:** Amino acid variation observed in CTV isolates^a^.

	p349-B aa at position:													
	
	9	19	165	214	228	230	246	250	254	259	284	290	291	51
	
M	L	R	S	A	Q	S	M	G	P	P	R	T	T	T
SC	V	P	HNV	T	RG	NI	V	N	SR	S	HW	A	AV	VIMN
T36			N	N	A	D		K	S		H	K	V	V
														

	p349-B aa at position:													
	
	84		103	119		142	143	169	173	186	205	221	222	233
	
M	R		W	L		F	H	R	S	N	S	K	T	S
SC	K		YSR	SND		VL	RT	GK	AT	D	RCH	GE	AE	P
T36			H	T		L	P	E	N		H	N	A	

														

	p349-C aa at position:				CP aa at position:			P13 aa at position:				P23 aa at position:		
				
	243		249	277		124			11		81		29	125

M	G		G	E		Y			I		S		K	E
SC	KR		S	VA		F			VXM		A		S	D
T36	R			D					M					

^a ^Predicted amino acid (aa) differences in five CTV genomic region among mild isolates (M), severe common isolates (SC) and T36 isolate. Only differences between T36 and SC are indicated for T36.

## Discussion

The genetic diversity of different regions on the genomic RNA of CTV isolates from Mexico was analyzed for the first time. The phylogenetic relationships of CTV have been analized utilizing genomic regions such as CP, p20, p23, p27, p349 (genomic regions A and F), 5'and 3' UTR.

To better understand the diversity among different CTV isolates from Mexico, genes CP, p23, p13, p349-B and p349-C were sequenced. This approach allowed the comparison of the Mexican isolates with those previously reported and also look for additional regions that may be useful to further characterize CTV strains. The regions analyzed here are located at opposite sides of the CTV genome.

To achieve homogeneity in the sequence alignments, the Mexican isolates were compared with six geographical and biologically distinct isolates and whose complete genome sequences are known: a severe quick decline isolate T36 from Florida-USA (Karasev et al., 1995), grapefruit stem pitting and a decline isolate VT from Israel (Mawassi et al., 1996), a sweet orange stem pitting and seeding yellow isolate SY568 imported into California-USA (Yang et al., 1999), a seedling-yellows-inducing isolate NUagA from Japan (Suastika et al., 2001) and two mild isolates, T385 from Spain (Vives et al., 1999) and T30 from Florida-USA (Albiach-Martí et al., 2000).

Our results showed that clustering of isolates highly correlates with their symptom severity. The overall branching pattern of the phylogenetic trees based on all regions were highly similar for all the mild isolates, including six of our Mexican isolates, forming a single cluster. The genetic distance analyses based on these segments indicated that the mild isolates were almost identical with a very low genetic diversity intra-group (lower than 0.022) independently from any analyzed ORFs. Such high similarity may be surprising since their considerable differences in geographical origin and time of sampling. This high similarity was observed for the first time dissecting the complete consensus sequences of the mild isolates T30 from Florida and T385 from Spain, which were geographically isolated for at least 24 years [12]. However, a different situation was observed for the severe isolates, where the genetic distance had a large range of variation (from to 0.066 to 0.276) and a unique branch could not be identified for them. In this way, the severe isolates were located more dispersed in the trees for all ORFs as opposed to the mild isolates.

The diversity among the severe isolates obtained was not random and the ORF p349, located in the 5'region of the viral genome was twice more variable than those located in the 3' end. The analyses of other two areas located into the ORF p349, region A (nucleotide 2021 to 2548) and region F (nucleotide 3561 to 3998), for 30 CTV isolates from Spain and California, showed appreciable differences because the former showed the greatest diversity (0.136). The values of genetic distance determined by us for the other two regions of p349:p349-B (nucleotides 1460 to 2350) and p349-C (nucleotides 6870 to 7900) were 0.143 and 0.169 respectively and coincide with those reported for the region A [11].

These observations were confirmed by comparing a sequence that covers the final 300 nucleotides of the p349-B region from 44 additional CTV isolates. This nucleotide stretch of DNA was found to be highly conserved in isolates from the same group (mean of 0.076), but highly variable among isolates of different groups (mean of 0.451), which supported the differentiation of CTV isolates for this region into three major genotypes here designed as: mild, severe common (all severe isolates excluding T36 group) and severe divergent isolates (T36-like isolates).

Sequences obtained from five Mexican isolates belonged to more than one group. It is possible that when a mixture of mild and severe clones are present in the host at different ratios, disease development can be restricted by a large excess of mild viral genome, even though the disease-causing variant remains at low levels in the viral populations. Previous reports have extensively demonstrated that most CTV isolates are composed by a population of genetically related variants (haplotypes) that could have originated from mixed infection of two or more CTV isolates with diverged sequence variants [13]. Also, the presence of mixed infection should be expected in woody plants that may live many years and are exposed to multiple aphid inoculations [14].

We observed that the nucleotide and amino acid variation were higher for 5' termini than for 3' termini indicating that the former target appears to be better suited to molecular clarification of relationship among CTV strains. In turn, the conserved 3' terminal nucleotide sequences would be insufficient to establish possible sub-groups inside of severe isolates to which the virus belongs.

Our results are in agreement with several reports that suggest a considerable degree of sequence variation in the CTV isolates mostly toward 5' termini of the genome. The sequencing of the 5' untranslated genomic region from eight CTV isolates from Spain and Japan, allowed their classification into three groups, I (severe-like T36), II (severe-like VT) and III (mild) represented by sequences of T36, VT and T317–8 (similar to T385), respectively, with a very low intergroup nucleotide identity ranged between 44 and 66%. This grouping was confirmed by sequencing an additional 15 virus isolates from several countries since all sequences could be assigned to one of the three types previously established [15].

Another report compared the predominant sequence variants of p23 gene from 18 CTV isolates of different geographic origin and pathogenicity (Sambade et al., 2003). As a result of the phylogenetic analyses, the CTV isolates were separated in three groups: first including the mild isolates, second including the most of severe isolates and third, an atypical group which showed variable pathogenicity. This last group was loosely related and included asymptomatic as well as seedling yellows isolates, maybe as result of recombination events (Sambade et al., 2003). In that report the intra-group genetic diversity for mild and severe isolates were similar of what we found, confirming that p23 is more heterogeneous that some others ORFs located toward 3' termini as p13 and CP. When we compared the eight Mexican isolates with those 18 reported for p23 [13], the divergence among the CTV isolates was supported for only two clearly distinct groups: mild and severe.

Similar mild genotypes have been found in several countries and led to speculate that this group is well adapted to its host and could have evolved from the same original sequence. As hypothesized, the divergences of the CTV strains could initiated in four possible different progenitors species in South East Asia prior to citrus cultivation [12]. According with this rationale, they spread to different citrus growing areas via infected budwood within the last 200 years. Then, mild strains could be distributed by growers due to the absence of symptoms on the commercial varieties and to the lack of certification programs.

In contrast, severe isolates induce stem-pitting, which affect the plant physiology and therefore reduce vigor and yield and cause eventually the death of the tree [2]. So, the visibly affected trees had been continuously eliminated or not propagated. Probably, like other viruses, the flow and evolution could have been interrupted several times creating successive bottlenecks leading to variability.

## Conclusion

This study represents the first attempt to analyze the genetic variability of CTV strains in Mexico. The analysis of eight CTV isolates, originally obtained from Mexican citrus fields, confirms that the CTV has been present in many citrus-producing of Mexico as a mixture of variants with different biological and genetic properties for long time. The divergence found between the isolates Mx-Tam and Mx-BC and also among these two severe isolates and the other six mild isolates plus the relative distant geographical separation of isolates, may suggest that it is unlikely that one strain evolved from the other after arriving to Mexico. It is likely, that the introduction of CTV to Mexico was under independent events an in several occasions to different citrus areas occurred via the use of infected propagative budwood sources.

Taking into account in the recent arrival of the aphid *T. citricida to Mexico*, the most important vector of CTV, and the predominance of sour orange as a rootstock in almost all the country, this fact should be of great concern for the future of the Mexican citrus industry.

The group-specific genome features identified here would provide a valuable tool for the generation of diagnostic tools for an early discrimination of virus strains based in at least five viral genome regions. Thus, the success of the citrus management strategies could depend in part, of the elucidation of the diversity and evolutionary relationships of the CTV isolates present in the different citrus areas of Mexico.

## Methods

### Virus isolates

Samples from CTV-infected plants were collected by technicians of the Dirección General de Sanidad Vegetal (DGSV), in eight citrus-growing areas of Mexico between 1983 and 2000. CTV isolates were graft-propagated and maintained in insect-proof greenhouses at DGSV headquarters at Mexico City. These isolates were biologically characterized on graft-inoculated indicator plants using different scion/rootstock combinations. Scions included Mexican lime, grapefruit, and sweet orange, whereas Citrus macrophylla and sour orange were used as rootstocks. Geographic origin and symptoms induced by these CTV isolates are summarized in table 5.

**Table 5:** Origin and biological characterization of eight Mexican CTV isolates.

CTV Isolates	Locality (State)	Date sampling	ML/CM		G, ML or SwO/SO	
			VC	SP	D	P
Mx-Yuc	Ticul (Yucatán)	2000	1	N	0	0
Mx-QR	Chetumal (Quintana Roo)	2000	1	1	0	N
Mx-Ver	Martínez de la Torre (Veracruz)	1986	1	1	0	0
Mx-Mich	Apatzingán (Michoacán)	1990	2	1	0	0
Mx-NL	Montemorelos (Nuevo León)	1994	2	1	0	0
Mx-Col	Tecomán (Colima)	1997	2	1	0	0
Mx-BC	Mexicali (Baja California Norte)	1997	3	2	0	0
Mx-Tam	Güemez (Tamaulipas)	1983	3	3	2	3

^a^Indicator species: ML, Mexican lime (*Citrus aurantiifolia*); CV; CM, *Citrus macrophylla; *SO, sour orange (*Citrus aurantium*); G, grapefruit (*Citrus Paradisi*); Sw, sweet orange (*Citrus sinensis*). ^b^VC, vein clearing and SP, stem pitting, D, decline; P, pinholing or honeycombing of the inner surface of the bark. ^c^Symptom intensity is scored from 1 to 3 with 1 being mild, 2 moderate and 3 severe.

### RNA isolation, reverse transcription and polymerase chain reaction (RT-PCR)

Total RNA was extracted using fresh bark tissue from healthy/CTV-infected plants in the presence of Trizol (Invitrogen, Carlsbad, CA) following the manufacturer's protocol. The final RNA preparation was dissolved in 30 μl of RNase-free distilled water.

For the reverse transcription (RT), 300 ng of total RNA and the reverse primer were incubated at 70°C for 5 min and chilled immediately on ice for 3 min. The mixture was added to provide a final concentration of 50 mM Tris-HCl, pH 8.3, 75 mM KCl, 10 mM DTT, 2.5 mM MgCl_2_, 500 μM of each dNTPs, 25 pmol of antisense primer, 20 U RNasin and 200 U MLV-RT (Promega, Madison, WI). This mixture was incubated at 42°C for 60 min. Resultant cDNA was then used subsequent PCR amplifications consisting of 10 mM Tris-HCl pH 8.8 at 25°C, 50 mM KCl, 2.5 mM MgCl_2_, 0.25 mM of each dNTP, 25 pmoles of each pair of primer and 10 units of Taq Polymerase (Promega, Madison, WI). PCRs were carried out in a iCycler Termocycler (BioRad, Hercules, CA) as follows: one cycle at 95°C for 2 min, then forty cycles at 95°C for 30 s, 50°C for 30 s, and 72°C for 1 min. In the last step samples were maintained for 5 min at 72°C.

Five different sets of primers were designed in our lab to direct the amplification of several genomic regions (table 6). These regions were selected as conserved regions after aligning the published CTV sequences from different geographical regions [8,12].

**Table 6:** Primers utilized in RT-PCR

Primer Name	Genomic region	Nucleotide sequence _(5' → 3')_	Primer localization (nt)^a^	Size of product (pb)
IRA 9	P349-B^c^	^5'^TTGGTTGGTGGTGAGTCTGC^3'^	1555–1574	876
IRA 10		^5'^GTGCCACTCGGAAAACTGAAAT^3'^	2414–2435	
IRA 11	P349- C^c^	^5'^TGAGCAGATCGGAGGTCTTG^3'^	6921–6940	1050
IRA 12		^5'^ACGTCATCGTCCAAATCCA^3'^	7952–7970	
IRA 1	CP	^5'^ATGGACGACGAAACAAAGAAATTG^3'^	16116–16139	672
IRA 2		^5'^GC TCAACGTGTGTTAA^3'^	16774–16787	
IRA 5	P13	^5'^GACTTAGACACGAAGTGACC^3'^	17250–17269	360
IRA 6		^5'^CTAAAGTAAGCTCGCATATTG^3'^	17721–17741	
IRA 7	P23	^5'^CGTGTAGGTTAATACGCTTCTC^3'^	18267–18288	630
IRA 8		^5'^CTTATTCCGTCCACTTCAATCAG^3'^	18982–19004	

^a ^numbering according to full-length of CTV isolate T385.

^b ^+ forward primer; - reverse primer

^c ^B and C are the regions of the ORF p349 that were analyzed in this job.

### Cloning and nucleotide sequencing

The reaction mixture was fractioned by agarose gel electrophoresis and the PCR products were purified using the GeneClean glass bead method (Bio101, Vista, CA) and ligated into pGEM-T Easy Vector System II (Promega, Madison, WI). The ligation mixture was used to transform *Escherichia coli *DH5α cells. Plasmids containing the expected sizes were chosen for sequencing. At least, three clones of each isolates were sequenced in both directions to obtain a consensus sequences by the Dye Cycle Sequencing kit (Applied Biosystem, Foster City, CA) in an ABI PRISM 377 sequencer

### Sequence alignment and phylogenetic analyses

Multiple sequences of different CTV isolates were aligned using the software MegAlign 4.0 of the Lasergene package (DNASTAR Inc. Madison). The unrooted and neighbour-joined phylogenetic trees were prepared using Clustal × [16] and drawn with TreeView 1.5 (Page, 1996). The robustness of individual internal phylogenetic tree nodes was estimated using 1,000 bootstrap reiterations. Clustal W algorithm was used for the estimation of genetic distance by the Kimura two-parameter method [17]. A genetic distance was also calculated using MEGA 2.1 with the output used to calculate both individual pairwise diversity and mean pairwise diversity using the Kimura-2-parameter approach.

The nucleotide sequences of the Mexican isolates obtained in this study were deposited in the GenBank database under the accession numbers: AF342890 to AF342895, AY649491 to AY649492, and AF652892 to AF652923.

Accession numbers for previously reported CTV nucleotide sequences used in this report are: T36 (U166034), VT (U56902), T385 (Y18420), SY568 (AF001623), T30 (AF260651), C268 (AJ599770), C269 (AJ579774), C270(AJ579771), T300(AJ579763), T305(AJ579776), T312(AJ579765), T32(AJ579766), T346(AJ579768), T388(AJ579777), T55(AJ579764), Barao (AJ579775), Cald (AJ579778), Val (AJ 579779), T405 (AF356250), T398 (AF356249), T373 (AF356248), T362 (AF356247), T346 (AF356246), T340 (AF356245), T315 (AF356244), T311 (AF356243), T309 (AF356242), T308 (AF356241), T300 (AF356240), 519 (AF356239), 416 (AF356238), 386 (AF356237), 384 (AF356236), 381 (356235), 379 (AF356234), 364 (AF356233), 162 (AF356232), 161 (AF356231), 143 (AF356230), G103 (AF356229), 59 (AF356228), 10 (AF356227), 5 (AF356226), 190 (AF203047), 173 (AF203043), 122 (AF203040), 107 (AF203037), 65 (AF203035).

## Competing interests

The authors declare that they have no competing interests.

## Authors' contributions

LHI carried out the molecular genetic studies, participated in the sequence alignment and prepared tables and figure. JCOS collected viral samples and organized information and material data. RRB and JPMS conceived of the study, and participated in its design and coordination. All authors read and approved the final manuscript.
